# Mercury in Feathers and Blood of Gulls from the Southern Baltic Coast, Poland

**DOI:** 10.1007/s11270-017-3308-6

**Published:** 2017-03-11

**Authors:** Emilia Szumiło-Pilarska, Lucyna Falkowska, Agnieszka Grajewska, Włodzimierz Meissner

**Affiliations:** 1 Department of Marine Chemistry and Environmental Protection, Faculty of Oceanography and Geography, University of Gdańsk, Al. Piłsudskiego 46, 81-387 Gdynia, Poland; 2 Avian Ecophysiology Unit, Department of Vertebrate Ecology and Zoology, Faculty of Biology, University of Gdańsk, ul. Wita Stwosza 59, 80-308 Gdańsk, Poland

**Keywords:** Mercury, Feathers, Blood, Gulls, Baltic Sea, Sentinels

## Abstract

Gulls were assessed as sentinels of contamination in the coastal zone of the Southern Baltic, research material being obtained from dead birds collected on Polish beaches and near fishing ports in 2009–2012. In feathers and blood of four gull species: herring gull (*Larus argentatus*), common gull (*Larus canus*), black-headed gull (*Chroicocephalus ridibundu*s), and great black-backed gull (*Larus marinus*), concentration of total mercury (Hg_T_) was assayed, taking into account the type of feathers, sex, and age. Stable isotopes (δ^15^N, δ^13^C) were used as tracers of trophic position in the food web. In the study, feathers and blood were compared as non-invasive indicators of alimentary exposure introducing mercury into the system. In order to do that, the correlations between mercury concentrations in the blood, feathers, and the birds’ internal tissues were examined. The strongest relations were observed in the liver for each species *R* ^2^_Common Gull_ = 0.94, *p* = 0.001; *R* ^2^_Black-headed Gull_ = 0.89, *p* = 0.001; *R* ^2^_Great Black-backed Gull_ = 0.53, *p* = 0.001; *R* ^2^_Herring Gull_ = 0.78, *p* = 0.001. While no correlation was found with feathers, only developing feathers of juvenile herring gulls were found to be a good indicator immediate of exposure through food (*R* ^2^_muscle_ = 0.71, *p* = 0.001; *R* ^2^_kidneys_ = 0.73, *p* = 0.001; *R* ^2^_heart_ = 0.89, *p* = 0.001; *R* ^2^_lungs_ = 0.86, *p* = 0.001; *R* ^2^_brain_ = 0.83, *p* = 0.001). Additionally, based on studies of herring gull primary feathers, decrease of mercury concentration in the diet of birds over the last two decades is also discussed.

## Introduction

Mercury is a toxic metal which accumulates in living organisms in the marine environment. Concentration of this element increases with trophic level. In result, organisms situated at the top of the trophic pyramid including birds, mammals, and humans are most exposed to toxic effects of mercury (Burger and Gochfeld 2004; Scheuhammer et al. 2007; Grandjean et al. 2010). Gulls feed in both land and marine ecosystems and therefore combine contamination from both. Many seabirds search for food in different areas, covering long distances, and hence, contamination of their tissues indicates pollution levels over a large area. Feathers, easy to collect in a non-invasive manner, are considered by many researchers to be good indicators of alimentary exposure and thus of environmental pollution with mercury (Burger 1993; Furness 1993; Ratcliffe et al. 1996; Furness and Camphuysen 1997; Thompson et al. 1998; Burger and Gochfeld 2004; Evers et al. 2008). Studies on mercury concentrations in feathers are widespread (Cairns 1997; Monteiro et al. 1999; Warner et al. 2010) and enable evaluation and comparison of mercury pollution in different areas of the world. Feathers are also useful in terms of monitoring environments in which birds and other organisms (including humans) live, as mercury present therein is both physically and chemically stable. Mercury concentrations in feathers of birds from museum exhibitions (Westermark et al. 1975; Thompson et al. 1993; Zolfaghari et al. 2007) allow us to determine concentrations of this metal in the previous century and identify long-term trend changes in environmental contamination. The second most popular form of non-invasive tissue research in birds uses their blood (Raygoza-Viera et al. 2013), which can reflect recent exposure to many substances (Barrett et al. 1996; Kahle and Becker 1999). The main entry routes of mercury into a bird’s body are ingestion of food and water, so quantity and quality of diet determine Hg body burden (Thomson et al. 1998; Cristol et al. 2008; Burger and Gochfeld 2004; Burger et al. 2009). Blight et al. (2015) found that seabirds from highly urbanized areas (Salish Sea, Canada) change the trophic level, moving increasingly towards food of anthropogenic origin. Therefore, in order to determine the trophic level and feeding area of wild birds, the studies were supplemented by an analysis of stable isotopes δ^15^N and δ^13^C (Hobson et al. 1994). In highly urbanized areas, absorption of the mercury by the birds’ respiratory system may be also important, but the problem is poorly described in the literature.

Research into the occurrence and accumulation of mercury in aquatic birds has been conducted for decades (Voitkevich 1966), particularly in North America and the Arctic (Stewart et al. 1997; Burger and Gochfeld 2004; Burger et al. 2009; Bond and Diamond 2009a, b), and demonstrated that, in addition to the ecological factor—diet, several biological factors including age, sex, molt pattern, and condition also helped to determine the concentration of mercury in birds.

In the Baltic, research into seabirds has tended to focus on Germany and Sweden (Westermark et al. 1975; Thompson et al. 1993; Thyen et al. 2000). Along the Polish coast of the Southern Baltic, no chemical monitoring of birds exists. Polish law prohibits the shooting of birds of the Laridae family but, with the permission of the Committee of Ethics, it is possible to gather information on the level of contamination in the habitat of these birds by sampling tissues of dead specimens and collecting feathers and blood from live ones.

Furthermore, only a few publications concerned with the Polish coast have focused on hazardous substances in the tissues of birds, namely the great cormorant (*Phalacrocorax carbo*), herring gull (*Larus argentatus*), mallard (*Anas platyrhynchos*), common merganser (*Mergus merganser*), and white-tailed eagle (*Haliaeetus albicilla*) (Falandysz et al. 1988; Misztal-Szkudlińska et al. 2011; Szumiło et al. 2013; Szumiło-Pilarska et al. 2015; Kalisińska et al. 2013; 2014a, b). Results for mercury concentrations in gull feathers are unavailable, except from Szumiło et al. (2013) and Szumiło-Pilarska et al. 2015.

The results presented in 2013 were based on 14 herring gull specimens and indicated a lack of significant differences between mercury concentrations assayed in soft tissues and feathers of specimens of various ages. That contradicted the data found in literature, a fact which encouraged the authors to extend the studies onto new material. In the paper by Szumiło-Pilarska et al. (2015), the earlier conclusions were confirmed. While showing no clear-cut differences between mercury concentrations in the soft tissues of 61 herring gull specimens of different ages, the study was extended to cover sex differences as well. The relationship between mercury concentrations in the soft tissues of four gull species: herring gull, common gull (*Larus canus*), black-headed gull (*Chroicocephalus ridibundu*s), and great black-backed gull (*Larus marinus*) was also analyzed. The lack of chemical monitoring of birds on the Polish coast, together with the restrictive law in Poland, caused the authors to lean towards non-invasive sampling based on feathers and blood.

The study aims were to compare the Hg concentrations in feathers of gull wintering in Poland and evaluate the relationship between Hg concentrations in feathers and soft tissues.

The authors wanted to know whether and under what conditions gulls could be considered good early warning sentinels of exposure threats to coastal communities, ecosystems, and humans. The studies looked at trophic level and type of feathers in four species of gulls: common gull, black-headed gull, and great black-backed gull. Sex and age differences were taken into account for herring gull.

Based on analyses of newly emerging covert feathers in herring gulls, an assessment of short-term contamination in the coastal foraging area is discussed. Herring gull primaries were used to determine the direction of changes in regional mercury contamination over the past two decades (1992–2012).

## Materials and Methods

The study used dead specimens of herring gull, common gull, black-headed gull, and great black-backed gull found between December 2009 and August 2012. Most birds were collected in an area of congregation near the fishing port in Wladyslawowo (N54° 47′, E18° 25′) and at “Mewia Lacha” bird sanctuary at the mouth of the River Vistula (N54° 21′, E18° 57′). A few specimens came from the beaches of the Gulf of Gdansk. In total, 104 birds were collected and this material was supplemented with 53 primary adult herring gull feathers collected in the same region in 1992–1993.

A bird’s age was determined on the basis of its feathers (Malling Olsen and Larsson 2004), and three age categories were established: juvenile, immature, and adult. In great black-backed gulls and herring gulls, juvenile birds were those in their first winter plumage (herrin gull, *n* = 24; great black-backed gull, *n* = 4), immature were in their second/third plumage (herrin gull, *n* = 14; great black-backed gull, *n* = 3), and adults were in their fourth/final plumage (herrin gull, *n* = 27; great black-backed gull, *n* = 3). With black-headed gulls and common gulls, juveniles were in their first winter plumage (black-headed gull, *n* = 4; common gull, *n* = 4), immature were in their second plumage (black-headed gull, *n* = 4; common gull, *n* = 12), and adult birds were in their final plumage (black-headed gull, *n* = 13; common gull, *n* = 3) (Szumiło-Pilarska et al. 2015). Owing to sexual immaturity and condition of internal sex organs, birds’ sex was subjected to PCR DNA amplification genetic testing (Fridolfsson and Ellegren 1999). During dissection, it was discovered that in most of the cases, the stomachs of birds were empty. Trophic level (δ^15^N) and the feeding area (δ^13^C) of the birds were therefore determined on the basis of studies on stable isotopes.

The number of samples was not equal to the number of birds, as it was impossible to isolate all types of feathers. The following feathers were collected from most specimens: outermost primary P_10_, innermost primary P_1_, rectrices, breast contour feathers, and down. Newly emerging covert feathers were also collected from nine herring gull specimens. All feathers were washed with 80% acetone in an ultrasonic bath, rinsed with Milli-Q water, and dried at room temperature. Whole feathers were used for analysis. Blood samples were taken, as a blood clot, from the bird’s heart, frozen (−20 °C), and lyophilized. Owing to the fact that that blood was collected from dead specimens, the wetness was not calculated and the results were given as dry mass.

During dissection, the cause of the birds’ death was not investigated, but in 9% of the cases, it was known (being run over by a car, freezing onto the ice layer, broken wing). The results assayed in the feathers of those specimens did not constitute a separate, distinguished set of data. Having compared them to information in literature on the subject of adverse effects, the authors believe that the level of mercury in the tissues of those birds could not have been the direct cause of their death. It was therefore concluded that the study was carried out on material that was representative of the study area.

### Chemical Analysis

Assay of Hg_T_ (total mercury) concentration levels was conducted by atomic absorption spectroscopy AMA-254. Mercury was determined in samples of dried biological material weighing 0.03 g (feathers) and 0.05 g (blood) (accuracy 0.0001 g). Each sample was placed in a prefired nickel boat and then automatically introduced into a furnace. There, the samples were dried at 120 °C for 300 s and mineralized at 550 °C over 180 s. Quills and vanes were analyzed thrice, and the final result represents an average of six analyses. Braune and Gaskin (1987) studied mercury concentrations in various parts of feathers and found no statistically significant differences between them. Mercury concentration results for blood samples are given as the mean of three replicates. Precision and accuracy of the Hg_T_ analysis method were gauged using CRM-BCR463 from the European Community Bureau ([Hg_T_] = 2.85 ± 0.16 mg kg^−1^), based on tuna. The average recovery rate for this material was 96.7% (Hg_T_), while the limit of quantification (LOQ) was 0.075 ng g^−1^ dry weight (d.w.).

### Stable Isotope Analysis

Analysis of stable isotopes δ^15^N and δ^13^C in the muscles and feathers of gulls was conducted using a Sercon 20-22 Continuous Flow Isotope Ratio Mass Spectrometer (CF-IRMS) coupled with Sercon SL elemental analyzer for simultaneous carbon-nitrogen-sulfur analysis. Weighted into tin capsules was 1.0 ± 0.2 mg of samples, and 0.5–0.75 mg of vanadium pentoxide was added as a catalyst. An in-house standard thiobarbituric acid (δ^15^N = +0.11 ± 0.9 (air), δ^13^C = −28.35 ± 0.06 (PDB)) was used as a reference. The results were expressed as differences in isotopic ratios as parts per thousand (‰) according to the equation:

$$ \delta X=\left[\left(\frac{R_{\mathrm{Sample}}}{R_{\mathrm{Standard}}}\right)-1\right]\cdotp 1000 $$

where *X* represents ^15^N or ^13^C and is the corresponding ^15^N/^14^N or ^13^C/^12^C ratio.

### Statistical Analysis

To assess the conformity of the distribution of Hg_T_ concentrations in biological samples with the normal distribution, the Shapiro–Wilk test was used. The significance of differences for two variables was analyzed using the Mann–Whitney *U* test, while the Kruskal–Wallis test was used for more variables. All results were ln-transformed to improve normality and stabilize variances. The relationship between Hg_T_ concentrations in the blood and other biological samples was defined using a coefficient of determination. To assess the quality of regression model applied, the root-mean-square error (RMSE) was calculated. These calculations used Hg_T_ concentrations for gulls’ soft tissues published by Szumiło-Pilarska et al. (2015). All statistical analyses were performed at a significance level of 5%. Statistical calculations and visualization of results were achieved using StatSoft Statistica 10 and Microsoft Excel 2007 with XLSTATA.

## Results

The results for mercury concentrations in various gull feathers and blood were above the detection limit, and their distributions did not conform with normal distribution in any case (Shapiro–Wilk test, *p* < 0.05). Of the four studied species, the great black-backed gull stood out in terms of Hg_T_ concentration levels, always demonstrating median concentrations at least two times higher than in the other species (Table 1). However, mercury concentrations in the various feather types did not indicate statistically significant differences between species (Kruskal–Wallis test, *p* < 0.05). Except for the innermost primary P_1_ and contour feathers, the lowest concentrations of mercury were determined in the black-headed gull.

**Table 1:** Statistical characteristics of Hg_T_ concentrations (ng g^−1^ d.w.) in different types of feathers from four gull species found on the Southern Baltic coast in 2009–2012

Species	Statistics	Outermost primary	Innermost primary	Rectrices	Contour	Down
Herring gull	*n*	57	54	55	61	33
	Median	736.6	1220.5	591.2	1525.9	1411.1
	x	1021.7	1640.9	1066.1	1910.7	1676.8
	RSD	301.3	434.0	339.0	407.0	386.7
	Min-max	40.1–6989.5	79.0–9186.8	62.4–6355.6	131.3–8211.9	450.2–4908.0
Common gull	*n*	17	19	18	16	8
	Median	621.1	584.0	609.9	1187.0	1548.4
	x	964.7	1037.3	1154.2	1322.9	1823.5
	RSD	451.7	479.5	684.0	574.3	1066.3
	Min-max	83.3–3006.4	106.8–3725.7	99.7–5481.4	124.0–4180.7	305.3–4062.3
Black-headed gull	*n*	18	19	19	19	14
	Median	523.2	897.3	380.4	1202.4	1033.0
	x	1344.1	1189.7	688.9	1549.0	900.2
	RSD	483.0	448.6	293.4	694.0	263.3
	Min-max	152.7–3323.1	165.3–3646.8	141.6–2196.2	325.2–7270.2	296.8–1767.9
Great black-backed gull	*n*	7	6	7	5	6
	Median	2273.4	2534.1	2464.8	3642.5	3249.6
	x	1879.7	3003.7	1705.1	3023.2	3213.7
	RSD	1027.4	1504.0	1122.0	1962.9	1468.4
	Min-max	141.6–2985.5	1204.0–5356.5	172.4–3046.5	187.9–4339.1	1361.1–5635.2

*n* number of samples, *x* mean value, *RSD* relative standard deviation

### Feathers from 1992 to 1993

**Fig. 1:**
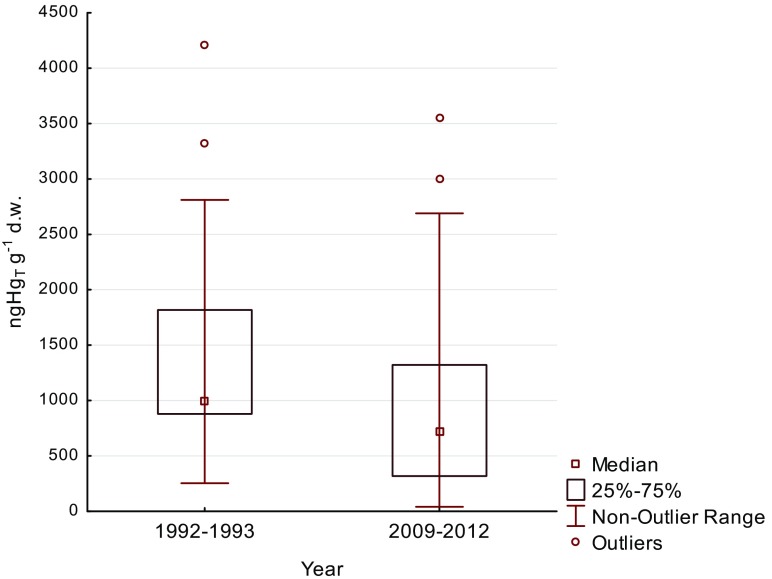
Mercury concentrations in herring gull remiges collected in 1992–1993 and 2009–2012 on the Southern Baltic coast of Poland

Median Hg_T_ concentrations noted in herring gull P_10_ remiges from 1992 to 1993 were higher compared to the same type of feathers from 2009 to 2012 (Fig. 1), the difference proving statistically significant (Mann–Whitney *U* test, *p* = 0.001). Only 16% of results from 1992 to 1993 were lower than the 2009–2013 remiges median. In order to improve the results’ transparency, the graph does not take into account extreme mercury concentrations in two 1992–1993 remiges 8587 and 8906 ng g^−1^ d.w. and one 2009–2012 remige 6990 ng g^−1^ d.w.

### Type of Feathers, Sex, and Age Differences in Mercury Level in Feathers

Analysis of the various feather types within each species showed no statistically significant differences in mercury concentration, except in herring gulls whose median mercury concentration in the innermost primary P_1_, contour feathers, and down was two times higher than the median for the outermost primary P_10_ and rectrices (Kruskal–Wallis test; *p* = 0.0001) (Table 1).

**Fig. 2:**
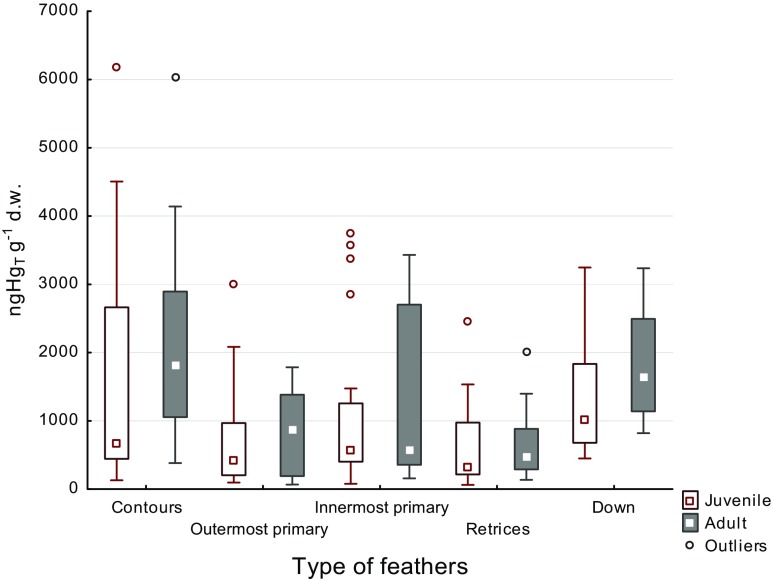
Box and whisker plot of mercury concentrations in different feathers in two age groups of herring gull found in the Gulf of Gdansk in 2009–2012. Note that extreme values (Table 1) are not shown in the figure

No statistically significant differences in Hg concentration were noted in any feather type between males and females of adult herring gull (Mann–Whitney *U* test). Almost significant statistical difference was found (Mann–Whitney *U* test; *p* = 0.060) only in contour feathers in two age groups: juvenile and adult (Fig. 2).

### New Contour Feathers

In developing covert feathers of herring gulls, the median Hg concentration was 2153.8 ng g^−1^ d.w, and results ranged from 399.5 to 4193.6 ng g^−1^ d.w. In this species, no statistically significant difference was observed between concentrations in fully developed and emerging covert feathers (Mann–Whitney *U* test, *p* = 0.3).

### Stable Isotopes

Stable isotopes δ^15^N and δ^13^C were determined in P_10_ remiges in all the studied gull species. The greatest differences in value, confirmed statistically by the Mann–Whitney *U* test (*p* = 0.01), were found between the common gull and great black-backed gull (+10.2 to +13.7‰ δ^15^N, respectively). The widest ranges for δ^15^N (+9.0 to +14.9‰) and δ^13^C (−24.9 to −18.6‰) were found for herring gull, while values for the common gull demonstrated the lowest dispersion (+8.7 to +10.9‰ δ^15^N; −23.8 to −22.3‰ δ^13^C).

### Hg_T_ in Blood

Of the four species studied, the highest average mercury concentration in the blood was determined in the great black-backed gull (median 1699 ng g^−1^ d.w.), while the lowest was in the black-headed gull (median 301 ng g^−1^ d.w.) (Table 2). Median Hg_T_ concentrations for the others were similar although a big difference was noted in common gulls between the median and arithmetic mean and high RSD, proving the high variability of mercury concentration in the blood. Moreover, with the common gull, two values were extremely high (3883.3 and 6087.4 ng g^−1^ d.w.) and on a similar level to the great black-backed gull.

**Table 2:** Statistical characteristics of mercury concentrations (ng g^−1^ d.w.) in the blood of four gull species found on the Southern Baltic coast in 2009–2012

Statistic	Herring gull	Common gull	Black-headed gull	Great black-backed gull
*n*	49	13	15	8
Median	525.1	459.8	300.9	1699.2
*x*	541.9	1163.5	515.4	2004.2
RSD	41.8	1062.4	324.2	1377.1
Min-max	15.4–1410.6	97.4–6087.4	21.3–2123.2	732.7–5987.5

Abbreviations as in Table 1

### Relationship Between the Concentrations of Hg_T_ in the Blood and Internal Tissues

A relationship between the concentrations of Hg_T_ in the blood and internal tissues (liver, kidney, muscle, heart, lungs) collected from the same individuals was established in all studied species (Table 3). The strongest relationship between Hg_T_ concentrations in the blood and internal tissues was observed in the common gull and the black-headed gull. A weaker relationship was noted in the herring gull and the great black-backed gull, with particularly low determination coefficients between the blood and the brain, heart, and lungs.

**Table 3:** Relationships between Hg_T_ concentrations (ln ng g^−1^ d.w.) in blood and soft tissues of gulls found on the Southern Baltic coast in 2009–2012 (*p* < 0.001 for all relationships)

Tissue	Liver			Kidney			Muscles			Heart			Lungs			Brain		
Species/age	*R* ^2^	*n*	RMSE	*R* ^2^	*n*	RMSE	*R* ^2^	*n*	RMSE	*R* ^2^	*n*	RMSE	*R* ^2^	*n*	RMSE	*R* ^2^	*n*	RMSE
Herring gull	0.78	39	0.294	0.66	39	0.468	0.60	39	0.555	0.46	39	0.611	0.59	37	0.482	0.52	35	0.474
Common gull	0.94	13	0.255	0.96	13	0.215	0.84	13	0.487	0.88	13	0.396	0.93	13	0.294	0.86	12	0.390
Black-headed gull	0.89	15	0.407	0.81	15	0.579	0.86	15	0.409	0.88	15	0.862	0.81	14	0.553	0.88	13	0.430
Great black-backed gull	0.53	8	0.547	0.73	7	0.282	0.57	8	0.610	0.38*	8	0.685	0.36	7	0.533	0.45*	8	0.488
Herring gull—juvenile	0.65	9	0.656	0.63	8	0.619	0.49	9	0.868	0.54	9	0.849	0.55	9	0.881	0.40*	7	0.618
Herring gull—immature	0.85	21	0.264	0.70	21	0.447	0.61	21	0.625	0.67	21	0.551	0.64	20	0.484	0.64	20	0.449
Herring gull—adult	0.77	17	0.329	0.80	17	0.413	0.52	17	0.537	0.66	17	0.501	0.72	16	0.445	0.73	15	0.414

*R* ^2^ coefficient of determination, *n* number of samples, *RMSE* root-mean-square error

*Statistically insignificant (*p* = 0.10)

Of the three age groups of herring gull, the relationships between the concentrations of Hg_T_ in the blood and internal tissues were similar for immature and adult specimens of herring gull (Table 3). In juvenile specimens, this relation was weaker and the strongest (*R* ^2^ = 0.65) occurred between Hg concentrations in the blood and in the liver. Differences between *R* ^2^ and RMSE values established in juvenile specimens and other groups were confirmed as significant by Student’s *t* test. No statistically significant differences in *R* ^2^ value between males and females of adult herring gull were noted (Student’s *t* test).

### Relationship Between the Concentrations of Hg_T_ in the Feathers and Internal Tissues

In birds from the Gulf of Gdansk, no correlations between the concentrations of HgT in the feathers and internal tissues were observed (*R* ^2^ value below 0.04), excluding new contour feathers of herring gull (Table 4). In this kind of feathers, the strongest relationship was observed for the heart (*R* ^2^ = 0.89), lungs (*R* ^2^ = 0.86), and brain (*R* ^2^ = 0.83).

**Table 4:** Relationships between Hg_T_ concentrations (ng g^−1^ d.w.) in new contour feathers and soft tissues of herring gull found on the Southern Baltic coast in 2009–2012 (*p* < 0.001 for all relationships)

Organ/tissue	*R* ^2^	*n*	RMSE
Liver	0.65	9	789
Kidneys	0.73	9	702
Muscles	0.71	9	725
Heart	0.89	9	441
Lungs	0.86	9	495
Brain	0.83	8	591
Blood	0.77	9	639

Abbreviations as in Table 3

## Discussion

### Long-Term Changes in Hg Concentration in Feathers

Historical material (gull feathers from museum collections) allowed Thompson et al. (1993) to present Hg_T_ concentration results, which reflected contamination of German territory over 150 years. That study suggested that the 1980s witnessed a drop in mercury contamination of Central Europe and, subsequently, a gradual reduction in Hg concentration in the feathers of birds residing along the German North Sea Coast. If Hg_T_ concentrations are compared in the down of chicks/juvenile gulls from two areas situated several hundred kilometers apart, in Germany (Becker et al. 1993) and Poland (Table 1), the concentration of Hg_T_ appears to have dropped by over 10% in 20 years. Changes in Hg levels along the Polish coast between 1992/1993 and 2009/2012 were apparent in the outermost primaries P_10_ of the adult herring gull (Fig. 1). The rate of decrease, assuming a linear change, was calculated at 1.5–1.9% year^−1^. In a similar period (1996–2011), monitoring at Mace Head (Ireland) showed an annual decline of 1.4–1.8% Hg^0^ in the air (UNEP 2013). A similar order of magnitude in the downward trend of mercury emissions (2.8% year^−1^) was established based on data from 11 Northern and Central European countries (UNEP 2013), and in Poland, following its social–economical transformation between 1989–2007, Hg emissions decreased by more than half. The trophic level determined in the outermost primary of the herring gull 20 years before (+12.8‰ δ^15^N) was close to the present level (+12.9‰ δ^15^N), so differences in Hg_T_ concentrations in feathers can be explained by a decrease in Hg emissions.

Comparison of mercury concentrations in feathers was based on recent findings in different regions. Gulls residing periodically on the Polish coast of the Baltic Sea demonstrated similar or lower Hg concentrations in their feathers, compared to gulls from other areas. In black-headed gulls from the Caspian Sea (Iran), Hg concentration in covert feathers and rectrices (5100–7100 ng g^−1^ d.w., Rajaei et al. 2010) was higher than in the same feathers of black-headed gulls from the Southern Baltic. In common gulls from the same area, Hg concentrations in covert feathers and rectrices were between 2090 and 2880 ng g^−1^ d.w., similar to values identified in this study. Mercury levels in feathers from the Caspian Sea fell within a narrower range when compared to the results of this study, possibly indicating higher alimentary exposure to mercury but less varied diet in birds from that region. In Greece in 2003–2009, mercury concentrations in chick feathers were between 825 and 1142 ng g^−1^ d.w. (Goutner et al. 2013), higher than juvenile herring gulls from the Polish coast. Feathers belonging to the great black-backed gull from the Polish coast were characterized by similar concentrations to those of the black guillemot (*Cepphus grylle faeroeensis*) from the Faroe Islands (Dam et al. 2004), while lower Hg_T_ concentrations were found in covert feathers of Olrog’s gull from Bahia Blanca (Argentina) (La Sala et al. 2011) and in remiges of adult Audouin’s gulls in Spain (Sanpera et al. 2007).

### The Effects of a Varied Diet

Many authors indicate significant differences in Hg concentration in feathers of birds which consume food from different trophic levels (Zolfaghari et al. 2007; Thompson et al. 1998; Furness et al. 1995; Monteiro et al. 1999; Bond and Diamond 2009b). Here, the median Hg concentration for the great black-backed gull was double that of any other species in all feathers (Table 1). An even greater range of species differences was evident in blood mercury concentrations (Table 2). However, the scant number of great black-backed gulls analyzed was probably responsible for the lack of statistically significant differences between mercury concentrations. Stable isotopes relating to diet and territory provide a more useful parameter. Great black-backed gulls grow feathers in Scandinavia and eat mainly foods from a higher trophic level (+11.6 to +16.2‰ δ^15^N; −17.4 to **−**21.0 δ^13^C) than other species scavenging further afield to include anthropogenic landfill sites (+8.7 to +15.3‰ δ^15^N; −18.61 to **−**24.87‰ δ^13^C). Probably, the composite diet of herring gull became the reason that there are no significant differences in the mercury concentration marked for individuals of different sex and age (Fig. 2). Professional literature points that the time of exposal is an important factor which has an impact on the mercury concentration in birds’ bodies (Furness and Camphuysen 1997). Nevertheless, the individuals nourished with a balanced diet have taken part in these examinations. The difference in the mercury concentration in individuals of different sex can be also observed in birds being nourished according to a constant diet.

Ninety years ago, Dwight (1925) found that each type of feathers in juvenile birds emerges at the same time, and therefore, mercury concentration in particular feather types is similar. Later research (Braune and Gaskin 1987) gave the same results. This study also confirmed previous reports. Median values of Hg_T_ concentration in particular feather types of juvenile specimens were similar. The only exception was down. In this type of feathers, higher mercury concentration was observed (Fig. 2). The relationships between mercury concentrations in small and large remiges (*R* ^2^ = 0.83; *p* = 0.01) have a curve inclination close to 1:1. However, in adults, the value of the coefficient of determination was lower (*R* ^2^ = 0.50; *p* < 0.05), and the trend indicated a greater increase in mercury with age in P_10_ remiges. Remiges are formed in specific order from 1 to 10. Mercury concentration results noted in the feathers of herring gulls and Bonaparte’s gulls (Braune 1987) demonstrated a statistically significant reduction in the concentration of mercury from the innermost primary I_1_ to the outermost primary P_10_. In the present study, the median concentrations of mercury in both types of feathers confirm the data from literature, although in ten individuals, there was a converse situation in which the mercury concentration in the outermost primary P_10_ (last to grow) was higher than that of the innermost primary P_1_ (first to grow). The cause of large differences between Hg concentrations, for example in the outermost primary I_10_ 4139 ng g^−1^ d.w. (+13.84‰ δ^15^N and −19.92‰ δ^15^C) and the innermost primary P_1_ 1750 ng g^−1^ d.w (+12.11‰ δ^15^N and −21.76‰ δ^15^C), is likely to be changes in food during the development of different feathers.

### Relationship Between the Concentrations of Hg_T_ in the Blood and Internal Tissues of Juvenile Herring Gull

It seems that in juvenile gulls, the fast gaining of weight (muscles, heart) and the delayed distribution of mercury with blood to accumulation sites account for determination of the relation at a lower level. In the group of juvenile birds, there were several-week-old chicks, whose organisms were burdened with mercury from the female laying eggs. The internal organs of a chick, particularly the muscles and the heart, as tissues of long-term deposition, reflect mainly mercury deposited in the egg and not that supplied with food (Burger and Gochfeld 1997). If the abundance of food frees birds from the necessity to use the energy accumulated as the fatty or muscle tissue, then the concentration of Hg_T_ in the blood reflects mainly alimentary exposure. The studied herring gull chicks were probably never stressed for food; therefore, the Hg concentration in their blood originates from an external source, that is food, which may be the main reason for a weaker dependence between Hg_T_ concentrations in the blood of chicks and in the muscle tissue.

### Feathers and Blood as Indicators of Environmental Pollution

The feathers of seabirds are considered by many scholars to be good indicators of pollution of coastal ecosystems (Thompson et al. 1993, 1998; Furness and Camphuysen 1997; Stewart et al. 1997; Mallory et al. 2010) as they are readily available, can be collected non-invasively, and, in contrast to blood, contain mercury, which is both chemically and physically stable. The existence of a relationship between mercury concentrations in feathers and internal tissues is proposed in numerous studies (Braune and Gaskin 1987; Thompson et al. 1991; Zamani-Ahmadmahmoodi et al. 2014). In birds from the Gulf of Gdansk, however, no such correlation was observed. Unlike in feathers, mercury in the gulls’ blood was largely correlated with the internal tissues in a statistically significant way (Table 3). Mercury is transported to feathers by blood during molting, which lasts several weeks (Lewis and Furness 1991; Dauwe et al. 2003), and its concentration in the blood is influenced by levels in food and body burden (Braune and Gaskin 1987). Studies on the Common Loon (Ontario, Canada) demonstrated a satisfactory relationship between Hg concentrations in its blood and in the fish on which it feeds (Scheuhammer et al. 1998). Monteiro et al. (1999) also demonstrated a strong relationship between Hg levels in the feathers of a bird of prey and the body of its prey. It was not possible to gather information on Hg concentrations in the Baltic gulls’ diets as the birds had empty stomachs. However, isotopic research of feathers and muscles (Szumiło-Pilarska et al. 2015) collected from the same individuals proved that Hg stored in this tissues may come from different sources (feathers +9.9 to +16.0‰ δ^15^N; muscle +8.3 to +12.1‰ δ^15^N). Thus, it is likely that no correlation exists between Hg concentrations in feathers and internal tissues. Ornithological observations agree with the results for stable isotopes, indicating large congregations of gulls near the fishing port and at the largest municipal landfill site in the coastal area (Gdansk-Szadolki) (Meissner et al. 2007).

**Fig. 3:**
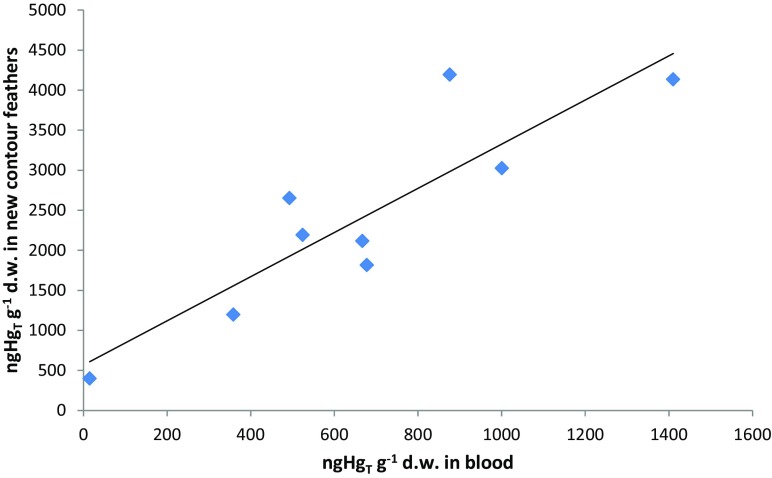
Linear relationship (*y* = 2.7597*x* + 565.65, *R* ^2^ = 0.77) between Hg concentrations in blood and developing covert feathers of herring gulls found on the Polish coast in summer 2011

Emerging covert feathers proved to be a good indicator of mercury concentration in blood and internal tissues (Table 4; Fig. 3.). A statistically significant relationship was also found between the Hg concentration in developing feathers and δ^15^N (*R* ^2^ = 0.71, *p* = 0.01). Migratory birds, such as the majority of gulls on the Polish coast, do not have a stable diet which, according to Furness et al. (1995) and Thompson et al. (1998), is key to determining long-term mercury contamination trends in migratory areas. On a short timescale (of days—Barregård 1993), Hg concentration results for blood may be a good indicator of environmental conditions (Table 3). Mercury in the blood can indicate both a dramatic environmental hazard and subtle, sublethal physiological responses to changes in habitat. Feathers develop over several weeks or longer and depend on availability of food (Verbeek 1977). Thus, feathers’ usefulness in evaluating contamination of the region where gulls eat increases. This applies particularly to the mature herring gulls as these birds breed on the Polish coast are mostly resident specimens (Neubauer 2011) and may therefore be more useful as environmental sentinels than other species. Birds migrating between Scandinavia, Western Russia, and Central Europe—black-headed gulls, common gulls, and great black-backed gulls—do not have a stable diet, but this does not preclude their usefulness as bioindicators of environmental contamination, as their habitat extends across the Baltic countries.

### Demethylation

**Fig. 4:**
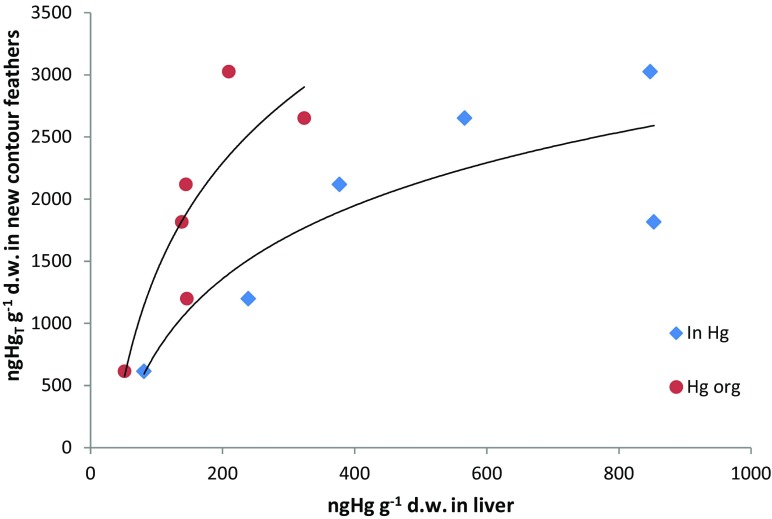
Relationship between total mercury in developing covert feathers and concentrations of Hg_org_ (*y* = 1269.6 ln(*x*) − 4436) and Hg_Inorg_ (*y* = 849.5 ln(*x*) − 3142) in the livers of herring gulls on the Polish coast in summer 2011 (results for organic and inorganic mercury concentration in the liver of the herring gull were taken from Szumiło-Pilarska et al. 2015)

Birds reduce mercury level in their bodies by removing Hg into developing feathers (Becker et al. 1994). The mercury in feathers constitutes about 95% body burden (Thompson and Furness 1989). Upon full growth of a feather, mercury contained therein is physiologically separated from the system (Voitkevich 1966). This route of removal (Becker et al. 1994; Braune and Gaskin 1987), along with demethylation, is the basic mechanism protecting birds from the most toxic form of mercury—methylmercury. Studies on birds from the Polish coast revealed a logarithmic relationship between Hg concentrations in developing feathers in the concentrations of organic mercury (Hg_org_) (*R* ^2^ = 0.73) and inorganic mercury (Hg_In_) (*R* ^2^ = 0.74) in the liver (Fig. 4). This indicated more effective demethylation in the liver, manifesting itself in a greater increase in inorganic Hg compared to organic mercury being incorporated into feathers at the same time. Mercury removal from the system may result from the features of a given species (Kim et al. 1996) or individual specimens (Bond and Diamond 2009b; Falkowska et al. 2013). Specimens with reduced possibility to demethylate use feathers as an alternative route of organic mercury removal. It was suggested that feathers can be indicators of demethylation efficiency (Bond and Diamond 2009b). Kim et al. (1996) observed, close to statistical significance, dependency between concentration of total mercury in feathers and a median of percentage MeHg content in the livers of seven gull species, including the herring gull from Northeastern Siberia.

## Conclusion

Migratory birds like the common gull, black-headed gull, great black-backed gull, and partially resident herring gull found on the Polish Baltic Sea coast migrate within an area which includes Northeastern Scandinavia, Western Russia, and Central Europe. Over/on that territory, they collect varied food, being the greatest environmental factor determining Hg levels deposited in a bird’s feathers and blood. Differences between age and gender were of low significance.

Differing levels and durations of alimentary exposure make it impossible to consider blood, mainly in mature gulls, and feathers as equal bioindicators of mercury contamination in birds’ bodies. Hg concentration in the blood enables effective tracing of local mercury contamination over a short time period of a few days (e.g., while laying eggs or feeding nestlings), while the feathers of mature gulls may indicate mercury contamination in the feeding area over a longer period. Gulls’ feathers can also indicate a long-term trend. Over 20 years (1992/1993–2009/2012) in the primaries of gulls from the Southern Baltic, there was a drop in mercury concentration. The results allow annual Hg concentration decrease to be calculated at 1.5–1.8%.

There is no chemical monitoring of birds on the Polish coast. However, the results collected over the 3 years suggest that adult herring gulls may be potential sentinels of environmental contamination with mercury on a local and regional scale, based on blood and developing feather tests. Newly emerging feathers can also indicate the effectiveness of demethylation in relation to fully developed feathers.
